# Study protocol: investigating the feasibility of a hybrid delivery of home-based cluster set resistance training for individuals previously treated for lung cancer

**DOI:** 10.1186/s40814-022-01065-5

**Published:** 2022-05-18

**Authors:** C. M. Fairman, O. L. Owens, K. L. Kendall, J. Steele, C. Latella, M. T. Jones, L. Marcotte, C. M. J. Peddle-McIntyre, K. K. McDonnell

**Affiliations:** 1 Department of Exercise Science, University of South Carolina, Columbia, USA; 2 College of Social Work, University of South Carolina, Columbia, USA; 3 School of Medical and Health Sciences, Edith Cowan University, Joondalup, Australia; 4 Faculty of Sport, Health, and Social Science, Solent University, Southampton, UK; 5 Department of Kinesiology, The University of Alabama, Tuscaloosa, USA; 6 Faculty of Medicine, University of British Columbia, Vancouver, British Columbia Canada; 7 Exercise Medicine Research Institute, Edith Cowan University, Joondalup, Australia; 8 College of Nursing, University of South Carolina, Columbia, USA

**Keywords:** Resistance training, Lung cancer, Cluster sets, Dyspnea, Fatigue, Physical function, Quality of life, Body composition, Feasibility

## Abstract

**Background:**

Symptom burden remains a critical concern for individuals with non-small cell lung cancer (NSCLC) following the completion of treatment. The most common symptom clusters, dyspnea (shortness of breath) and fatigue, can contribute to physical decline, reductions in quality of life, and a higher risk of comorbidities and mortality. Dyspnea is a primary limiter of exercise capacity in individuals with lung cancer, resulting in exercise avoidance and an accelerated physical decline. As such, designing resistance training with cluster sets to mitigate symptoms of dyspnea and fatigue may result in improved exercise tolerance. Thus, maintaining the exercise stimulus via cluster sets, combined with improved tolerance of the exercise, could result in the maintenance of physical function and quality of life. The purpose of this study is to investigate the feasibility and preliminary efficacy of a hybrid-delivery home-based cluster-set resistance training program in individuals with NSCLC.

**Methods:**

Individuals with NSCLC (*n* = 15), within 12 months of completion of treatment, will be recruited to participate in this single-arm feasibility trial. Participants will complete 8 weeks of home-based resistance training designed to minimize dyspnea and fatigue. The hybrid delivery of the program will include supervised sessions in the participants’ home and virtual supervision via video conferencing. The primary outcome of feasibility will be quantified by recruitment rates, retention, acceptability, and intervention fidelity. Exploratory outcomes (dyspnea, fatigue, quality of life, physical function, and body composition) will be assessed pre- and post-intervention.

**Discussion:**

This study will provide important data on the feasibility of delivering this intervention and inform procedures for a future randomized controlled trial.

**Trial registration:**

Record not yet public

## Background

Advances in the detection and treatment of lung cancer have resulted in an increasing number of individuals living beyond treatment [1]. Unfortunately, individuals with lung cancer are also burdened by clusters of symptoms, most typically dyspnea (shortness of breath) and fatigue [2, 3]. These symptoms contribute to reductions in physical activity and an accelerated trajectory towards physical disability, placing individuals at a heightened risk of developing or exacerbating comorbidities such as chronic obstructive pulmonary disease and cardiovascular disease [4, 5]. In particular, symptom clusters of dyspnea and fatigue have been recognized as a contributor to the reduction in exercise capacity in individuals with cancer [6, 7]. Importantly, poor exercise capacity is associated with exercise avoidance, functional decline (i.e., worse 6-min walk test score), poor prognosis, and lung cancer mortality [8]. Consequently, there is increasing emphasis on exercise capacity as an important outcome in the management of the disease [9].

The configuration of resistance training (RT) sessions may be designed in a way to mitigate symptoms of dyspnea and fatigue, while maintaining sufficient RT stimulus to improve exercise capacity in individuals with lung cancer. Traditionally, RT prescription for individuals with cancer has included multiple sets of consecutive repetitions ranging anywhere from 6 to 15 [10]. It has been suggested that performing consecutive repetitions may result in excessive fatigue, and thus perception of fatigue, which may negatively impact affective responses and motivation for exercise training [11]. Given the debilitating effects of dyspnea in individuals with lung cancer and the proposed influence of dyspnea on exercise capacity, there is a strong rationale to investigate strategies aimed at minimizing dyspnea during exercise in this population [6].

Cluster sets generally refer to the inclusion of intra-set rest periods (i.e., additional rest during the set itself) [12]. Essentially, cluster sets may allow for reduced levels of fatigue at similar loads of training or enable individuals to perform a greater volume/quantity of training with similar levels of fatigue. Prior research demonstrates that RT with intra-set rest results in the attenuation of fatigue that accompanies consecutive repetition protocols (when training loads are equated), resulting in lower perceptions of effort in comparison to traditional configurations [13–15]. Thus, there is potential for cluster sets to result in lower levels of dyspnea and fatigue, which could facilitate greater exercise tolerance and adaptations in physical function and symptom clusters in individuals with lung cancer.

Home-based exercise is a promising strategy to overcome traditional barriers of facility-based exercise interventions (e.g., resources, travel, cost, limited hours) [16]. We hypothesize that a hybrid approach of delivering exercise programming both in-person and through video conferencing (i.e., live delivery of program via video conferencing) may address some of the anticipated barriers. The initial presence of an exercise professional could help with the delivery of safe and appropriate exercise, whereas the transition towards a hybrid approach could foster independence and autonomy. Furthermore, the impact of COVID-19 has necessitated creative solutions to traditional in-person training, whereby the hybrid delivery approach can help ensure safe exercise, but also limit risk in a potentially vulnerable group [17].

The aims of this study are to (1) conduct a single-arm study to evaluate the feasibility of a hybrid delivery home-based RT program by measuring recruitment, retention, acceptability, and intervention fidelity among survivors and (2) gather data using a pre-/post-test design, to estimate preliminary intervention effects on (a) reducing sensations of dyspnea and (b) improved fatigue, physical function and body composition, and quality of life among survivors immediately post-intervention.

## Methods/design

**Fig. 1:**
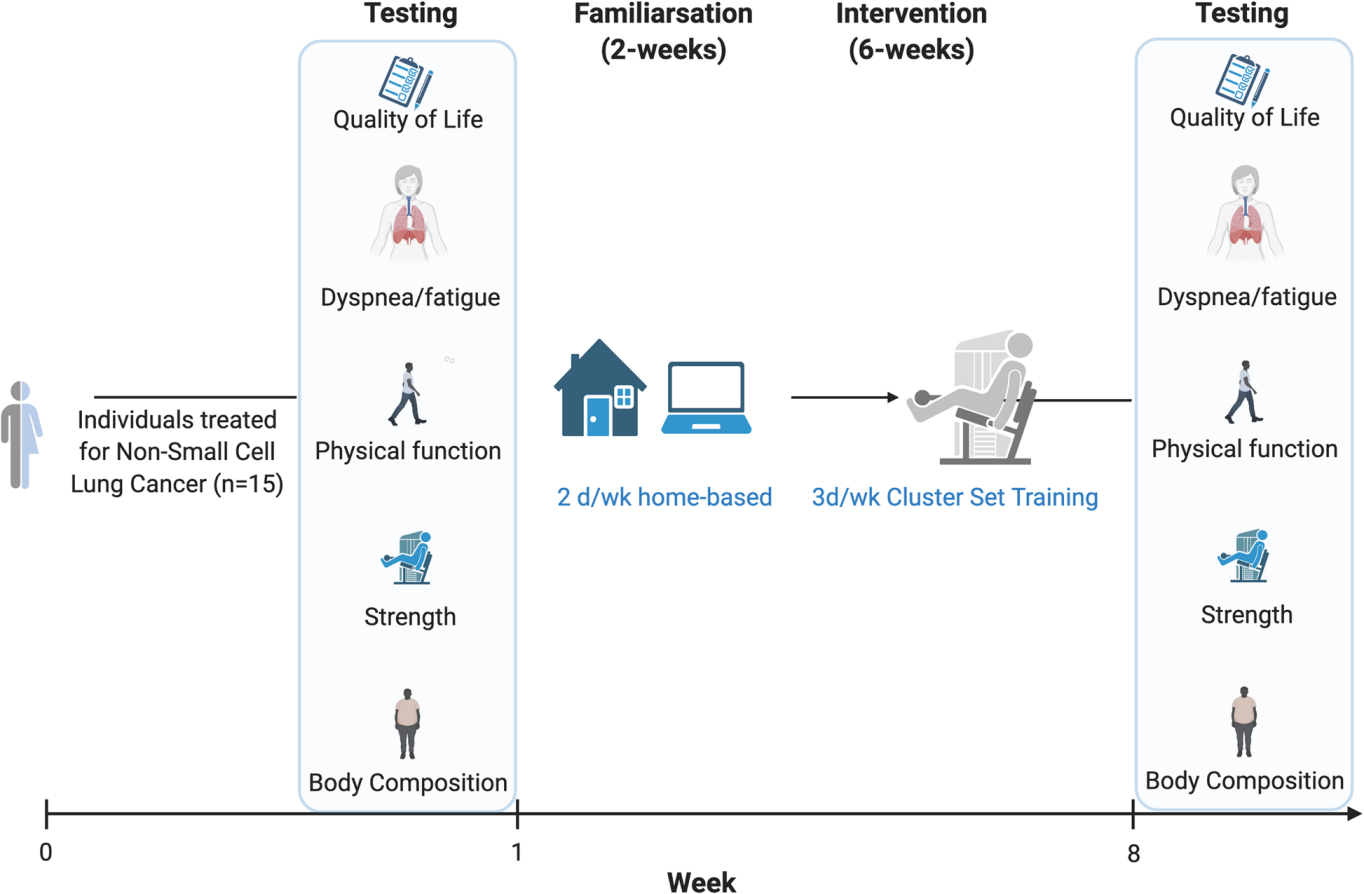
Overview of the study design

This is a single-arm feasibility trial evaluating the effects of an 8-week hybrid delivery of RT with individuals treated for NSCLC. The primary outcome of feasibility will be assessed via recruitment, retention, and intervention fidelity (outlined below). Additional outcomes include health/wellness (dyspnea, fatigue, quality of life), muscular strength (5 repetition maximum and 5 times sit-to-stand), body composition (dual-energy X-ray absorptiometry), and exercise capacity (6-min walk test). Outcomes will be assessed pre-/post-intervention. A convenience sample (*n* = 15) will be recruited to determine the feasibility of the intervention. The sample size was determined based on the recommendations from Julious et al. that justify a minimum of *n* = 12 based on feasibility, gains in precision surrounding the mean and variance, and ability to estimate parameters for future studies [18]. Consequently, in line with other exercise oncology trials and in anticipation of an ~75% retention rate, we aim to recruit 15 individuals to participate. The trial is under review at clinicaltrials.gov and deviations to the protocol or trial procedures will be reflected there and reported in the final manuscript. This study is approved by the University of South Carolina’s Institutional Review Board (Pro00110261). An overview of the study scheme is presented in Fig. 1.

### Pre-established criteria for success

The feasibility of the intervention will be determined based on the following:

1. Recruitment: The recruitment goal of *n* = 15 in 1 year has been reached.
2. Retention: If ≥75% of the sample recruited to participate return for follow-up testing.
3. Intervention fidelity: If relative dose intensity (RDI) (outlined below) is ≥70%.

Intervention fidelity will be reported using metrics previously reported by Fairman et al. [19]. Specifically, volume load will be calculated as the product of the number of sets x repetitions for each exercise and summed to give the total volume for each session. We will report the proportion of volume achieved relevant to what was prescribed to give a RDI for each person. RDI will then be averaged and used to determine fidelity to the RT intervention.

The study team will evaluate the feasibility and exploratory outcomes to determine if progression to a randomized controlled trial is warranted. Specifically, quantitative information from focus groups (outlined below) will be contextualized, along with qualitative data on the feasibility to provide greater insight into participant experiences and determine the continuation for a full trial [20]. Additionally, unforeseen logistical challenges regarding intervention delivery will be documented, managed, and modified as necessary if the decision to move forward to a randomized controlled trial is made.

### Participant recruitment and screening

Recruitment will occur in collaboration with a medical and radiation oncology practice (South Carolina Oncology Associates [SCOA]) closely affiliated with an American College of Surgeons (ACOS)–approved Integrated Network Cancer Program at a large community hospital in central South Carolina. Participants will be recruited by (1) use of cancer registry databases at local health care system’s cancer programs and (2) outreach with various cancer support groups and organizations.

Specifically, a partial waiver of authorization is approved by the local Institutional Review Board so the research team can mail an invitation—a one-page, culturally sensitive postcard that includes a study telephone number and email address (to RSVP or get more information about the study)—to all potential participants identified through the cancer registry database. In the event that recruitment numbers are insufficient, we will work closely with our clinical partners (medical, pulmonary, and nurse navigators) who have pre-established relationships with potential participants. The staff of these offices will recruit potential participants in person as they encounter them in their daily practice. A third recruitment method will use the same invitation from the primary recruitment method; instead of being mailed directly to potential participants, the postcard will be positioned in select physician offices and clinics as well as distributed to support groups and programs for individuals with lung cancer.

Moreover, we will closely monitor the response to mailings and adjust our efforts accordingly. Specifically, mail flyers and invitations will be mailed in batches of 100 potential participants. We will schedule mailings to occur approximately every 4–6 weeks until recruitment goals are met. If recruitment numbers are insufficient, we will increase both the size and frequency of mailings.

Within a week of the first anticipated invitation arrival, telephone contact will be initiated with the recipients by a member of the research team. Over the phone, using a script, the research specialist will screen individuals indicating interest in participating to determine eligibility. Additional follow-up telephone contact will be made after the individual has had adequate time (~1 week) to make a decision and speak with a family member about participating. These telephone interactions are designed to strengthen trust in the research team, build credibility, and increase understanding of the study. During this call, interested individuals will be provided with detailed information about the study purpose/aims, protocol, potential benefits, and risks and be asked to answer further eligibility questions. Individuals who are eligible and remain interested will be asked to provide verification of medical clearance and provide informed consent to participate prior to any study activities.

Pre- and post-intervention data collection will take place at the University’s Department of Exercise Science. Exercise sessions will take place in participants’ homes or other convenient locations. Informed consent will occur prior to baseline testing, where a member of the study team will review the consent form in detail and answer any further questions participants might have. Every effort will be made by study staff to ensure the informed consent is understood in its entirety prior to signing.

### Participants

Individuals diagnosed with NSCLC stages I–III who have completed their primary cancer treatments within 12 months and have obtained a medical clearance (from a general practitioner or medical team) to engage in RT will be recruited. Participants will be excluded if they (1) have a diagnosis of advanced (stage IV) lung cancer or diagnosis of small cell lung cancer; (2) are not comfortable having study staff visit their homes for RT sessions; (3) have any neuromuscular, cardiovascular, or psychological condition (assessed using self-report questions during eligibility screening) precluding safe exercise; (4) have participated in structured RT ≥ 2 times/week for the past 6 months; or (5) are unable to read/understand English. Further description of eligibility criteria is provided in Table 1.

**Table 1:** Eligibility criteria

**Inclusion criteria**	• Completed definitive treatment for localized NSCLC (stages I–III) within 12 months of completion • Has access to stable Internet access for Zoom participation • Willing to complete an 8-week, home-based intervention program that includes face-to-face and Zoom interaction • Willing to consider behavior change at this time • Able to speak and read English • Capable of informed consent • Has obtained medical clearance from a medical practitioner or medical team
**Exclusion criteria**	• Individuals with a known diagnosis of advanced lung cancer (stage IV; due to potential added burden) or diagnosis of small-cell lung cancer • Anyone for whom physical activity is not recommended • Are not comfortable having study staff visit their homes for exercise sessions • Have any neuromuscular, cardiovascular, or psychological condition precluding safe exercise • Have participated in structured RT ≥2 times/week for the past 6 months • Are unable to read/understand English

### Intervention

The hybrid RT program will include a combination of in-person and virtual delivery of live exercise sessions (Table 2). The intervention will be delivered by study staff specifically trained in the delivery of exercise programs for individuals with cancer. The first 2 weeks will include a member of the study team visiting the home of participants (or other mutually agreed upon private location) two times per week to deliver supervised exercise sessions. The purpose of this phase is to (1) determine a safe place to exercise in the individual’s home or mutually agreed upon, private location and (2) provide in-person instruction on safe exercise technique and general procedure for exercise sessions (sets, repetitions, clusters, anchoring of perceived exertion/dyspnea scales, etc.). Selection of exercises and loading will be based on each individual’s strength levels, mobility, and any physical limitations. Participants will be provided dumbbells, kettlebells, and suspension training bands (TRX, San Francisco, USA) to complete the home-based exercises. In addition to exercise selection and the delivery of exercise sessions as above, the initial 2 weeks of the study period will also be used to guide participants on how to use the computer tablets, log in for exercise sessions, and record their exercise participation/RDI. Moreover, written instructions in plain language will be provided. Tablet stands will also be provided to allow for more flexibility regarding the placement of tablets for easy viewing.

**Table 2:** Overview of hybrid delivery approach for the program

	Weeks																					
	1		2		3			4			5			6			7			8		
Video					X	X	X	X	X		X	X	X	X	X		X	X	X	X	X	
In person	X	X	X	X						X						X						X

The exercise sessions will include a warm-up, “main session” including six total movements targeting total body musculature (hip hinge, squat, horizontal push, vertical push, horizontal pull, trunk), and a cool-down period. The exercise program is designed acknowledging some of the limitations of home-based exercise and equipment availability. Moreover, the protocol will initially be piloted on 2–3 individuals prior to the trial. Specifically, the study staff will identify several individuals who are representative of the target study population. These individuals will undergo the same eligibility screening as program participants. If deemed eligible, participants will be asked to come to the laboratory at the University of South Carolina to pilot the program, in line with procedures from a recently published study on designing home-based exercise programs among older adults [17]. Individuals will take part in the exercise program on two separate occasions. Individuals will then be asked to provide experience on all aspects of the program (level of technicality, perceived level of difficulty, safety concerns, and potential challenges). This feedback will be used to finalize the exercise protocol for the trial. The final exercise protocol will be presented in its entirety in future publications.

Sessions will be progressed by adding sets/repetitions to the exercises and increasing load as appropriate and tolerated (i.e., in exercises where no load can be added, a focus will be on adding sets or changing body position to adjust moment arm lengths). Specifically, trained staff on the trial will select the appropriate exercises, provide coaching cues for safe movement, and tailor exercise sessions for each individual. To assist with transparency and reproducibility, de-identified training logs including exercise selection, sets, repetitions, and load will be made available on an open access repository after completion of the study, in line with other exercise oncology studies [21]. Following the 2-week in-person period, participants will be asked to complete 3 sessions per week using live, virtual sessions. These sessions will be delivered in a ratio of up to 4:1 of participants to instructor. Every sixth session, the exercise session will be delivered in-person to address any concerns, provide modifications if necessary, and ensure safe progression of exercises. Individuals will be given the option of a paper- or tablet-based (using tablets provided) program to record aspects of intervention fidelity (sets, repetitions, and weight), dyspnea with exercise, and perception of effort. Reasons for missing sessions will be recorded. Missed sessions will not be made-up through extra sessions during the 8-week training period.

### Study outcomes

Study outcomes will be assessed within 1 week of initiation and at program completion. The exit interview will be conducted within 2 weeks of program completion. An overview of assessments is provided in Table 3.

**Table 3:** Overview of testing and timeline of study activities

Outcomes	Baseline	Weeks 1–8	Post-testing
Informed consent, medical history, and demographics	X		
Feasibility outcomes: recruitment, retention, fidelity, acceptability	X	X	X
Health/wellness questionnaires: dyspnea, fatigue, quality of life	X		X
Body composition: dual-energy X-ray absorptiometry (DEXA)	X		X
Muscular strength: 5 repetition maximum	X		X
Physical function: 6-min walk test and 5 timed sit to stand	X		X
Exit interview			X

### Outcome measures

The primary outcome of feasibility will be evaluated by (1) recruitment (successful recruitment of target sample size, *n* = 15, in 1 year); (2) retention (number of participants who complete follow-up assessments, with a threshold set at ≥75%); (3) intervention fidelity (proportion of exercise completed, relative to what was prescribed, with ≥70% considered successful) [19]; and (4) intervention fidelity: if relative dose intensity (RDI) (outlined above) is ≥70%.

#### Acceptability

Intervention acceptability will be assessed using a 10-item questionnaire adapted from McDonnell et al., assessing the acceptability of intervention components on a 4-point Likert-type scale from “Strongly disagree” to “Strongly agree” [22]. Specifically, individuals will be asked about the ease of use of the tablet, utility of virtual exercise sessions, and level of personalization of the program. Additionally, individuals will be asked to participate in an exit interview at the end of the study to learn about their experiences and opinion of the program. Focus groups will be inductive in nature and allow participants to elaborate on their experiences and perceptions of the program. Interviews will be recorded, transcribed, and coded for thematic analysis (nVivo Software, QSR International, Burlington, MA, USA).

#### Dyspnea

Dyspnea will be assessed using the FACIT-Dyspnea (FACIT-D) 10-Item short form [23]. The FACIT-D is divided into two, 10-item scales that assess the level of dyspnea experienced with different activities (part 1) and the difficulty performing various activities as a result of dyspnea in the preceding 7 days (part 2). The scale is scored on a Likert-type response from “no shortness of breath” to “severely short of breath” (part 1) or “no difficulty” to “much difficulty” (part 2).

#### Fatigue

The FACIT-Fatigue scale is a 13-item scale that will be used to assess cancer-related fatigue [24]. The FACIT-Fatigue scale is scored on a 0–4 response scale from 0 = “not at all” to 4= “very much,” regarding items related to fatigue and energy in the past 7 days.

#### Quality of life

Quality of life will be assessed using the Functional Assessment of Cancer Therapy-Lung Cancer Subscale (LCS) [25]. The LCS is a 9-item scale assessing multidimensional aspects of quality of life relating to lung cancer. Items are scored on a 5-point Likert scale from 0 = “not at all” to 4 = “very much,” anchored to the past 7 days.

#### Five-repetition maximum

Muscular strength will be assessed using five-repetition maximum (5RM) testing for leg press and chest press exercises. These are standard tests commonly used in exercise oncology trials [19, 26, 27]. Participants will be asked to perform a general warm-up, followed by two exercise-specific warm-up sets (4–6 repetitions) with increasing load, separated by 90–180s of rest. Participants will then be asked to complete a 5RM attempt (the amount of weight an individual can lift with proper technique through a full range of motion). A 5RM score will be determined when the participant can no longer complete 5 full, safe repetitions of each exercise. In this case, the last load completed safely will be recorded as the 5RM [19, 26, 27]. Approximately 3 min of rest will be provided between attempts and efforts will be made to ensure a 5RM is reached in as few attempts as possible.

#### Physical function

Physical function will be assessed via the 5 times sit-to-stand and the 6-min walk test(s). The 5 times sit-to-stand will be conducted by recording the time (in seconds) taken to stand 5 times from a sitting position as quickly as possible. The 6-min walk test will consist of recording the total distance traveled on a flat surface at a self-determined pace in a 6-min period [28].

#### Body composition

Body composition will be assessed via dual-energy X-ray absorptiometry (DEXA) [29]. Assessments of the whole body and appendicular lean mass, in addition to fat mass and bone mineral content, will be obtained. Appendicular lean mass from DEXA and score on the 5 times sit-to-stand will be used to quantify the proportion of individuals who are sarcopenic at pre- and post-intervention, respectively [30].

#### Session effort and dyspnea

Ratings of perception of effort and dyspnea will be recorded for each exercise session. Individuals will be prompted by study staff after every exercise session to record ratings. Specifically, the rating of perception of exertion – effort (RPE-E) scale will be used to assess each participant’s ability to meet the demands of the exercise session [31]. The RPE-E is a single-item scale score from 0 = “No effort” to 10 = “Maximal effort.” Overall dyspnea experience during the session will be assessed using the modified Borg scale-dyspnea (MBS-D). The MBS-D is a scale labeled 0–10, asking participants to rate the best description of their shortness of breath (0 = “Nothing at all” to 10 = “Maximal”) [32, 33]. Participants will be familiarized with scales during the initial study home visits and will be clearly instructed to differentiate these two perceptions when providing ratings.

### Adverse events

The risk of exercise-related adverse events will be minimized by the initial supervision, exercise selection, and technique correction from trained exercise physiologists on the trial. However, we will record and report any adverse events in accordance with the Consolidated Standards of Reporting Trials guidelines. A trial steering committee (CMF, SO, KKM) will oversee the trial and will be responsible for auditing procedures, in addition to recording and reporting any adverse events that arise. Due to the low risk of adverse events in exercise trials and that individuals in the trial will not be on active treatment, a data management committee was not assigned. Adverse events will be recorded and reported as required by the Institutional Review Board. Data including a description of the event, its relation to the intervention (i.e., not related, unlikely, possibly, probably, definite), its level of seriousness (i.e., non-serious, required hospitalization, resulted in persistent disability, life-threatening, or resulted in death), and its intensity (i.e., mild, moderate, severe, life-threatening) will be collected. Additionally, the participant will be referred to their general practitioner or specialist as appropriate for a medical assessment of any adverse event. Participants are free to withdraw from the study at any time.

### Data management

Data collected will be de-identified and coded. Each study participant will have a unique study identification code created following informed consent comprised of two random letters and a consecutive number. Electronic data will be kept in a folder on a password-locked computer in the Department of Exercise Science at the University of South Carolina and only accessible by the investigators and designated research staff. Survey data will be collected using a password-encrypted, online data collection system (RedCap). At the completion of every data collection session, any physical documentation will be stored in a secure filing cabinet with restricted access in a private office in the Department of Exercise Science University of South Carolina. All information will be held in these secure locations (with password protection or key-lock access) and will not be stored external to the university. All paper-based records will be stored in a locked filing cabinet with restricted access in a private office for a minimum of 7 years.

Participants will be provided with an explicit description of plans to share any data they contribute, and how their privacy and confidentiality will be protected. All de-identified data and code for analyses will be made available as soon as possible with publication of the primary outcome paper. The dataset will include demographic information and physiological and psychosocial measures outlined in the proposal. We will make the de-identified data and associated documentation available to users only under a data-sharing agreement that provides a (1) commitment to using the data only for research purposes, (2) commitment to securing the data using appropriate computer technology, and (3) commitment to destroying the data after analyses are completed.

### Statistical analyses

We will use descriptive statistics (percentages and means [with standard deviations]) to report on feasibility outcomes (e.g., recruitment, retention, fidelity). Means and standard deviations will be reported for assessments of exploratory outcomes at baseline and follow-up [18]. Baseline and follow-up testing will be conducted on a per-protocol basis. Descriptive statistics of secondary outcomes will be reported and used to inform sample size calculations for a future randomized controlled trial and aid in decisions on whether their inclusion in future trials is warranted [18, 34]. Analysis will be performed with raw data and code shared on osf.io.

### Limitations

The study has several limitations worthy of addressing. Notably, we are not recruiting individuals with lung cancer with documented dyspnea and fatigue. Recruiting individuals who have reached a certain threshold of dyspnea and fatigue would be a considerable strength of the study in understanding the feasibility and impact of exercise in individuals with documented impairments. However, we opted against this for several reasons. Firstly, there is limited evidence regarding the feasibility and/or effectiveness of exercise in lung cancer in general [35]. Moreover, there is evidence of RCTs of exercise in lung cancer being terminated due to slow accrual and difficulty recruiting individuals with lung cancer to exercise [36]. For those reasons, we opted for an intentionally broad recruitment pool for this feasibility stage of trial development. The inclusion of individuals who are below a certain threshold for dyspnea is something we will be strongly considering for the next stages of our trial development, should this trial be successful.

### Dissemination

The findings of the proposed study will be published in peer-review journals and presented at relevant national and international scientific meetings. Furthermore, the study findings will be translated into plain language and presented to all participants in addition to interested community partners associated with the investigative team.

## Discussion

The primary purpose of this trial is to assess the feasibility of a hybrid delivery of cluster set RT for individuals previously treated for NSCLC. Publication of this protocol is to enhance the transparency around trial success, outcomes of interest, and to aid in the replicability of our protocol.

Progress in improving exercise capacity and exercise participation in this complex clinical population is likely to be supported through individualized approaches that are patient-focused. This single-arm study will examine the intervention feasibility in individuals with NSCLC. Novel aspects of this trial include (1) the investigation of a hybrid delivery of exercise prescription, combining supervised, home-based sessions and distant video-conferencing sessions, and (2) the inclusion of a method of exercise prescription specifically aimed at reducing symptom burden in NSCLC. Despite the potential applications and health benefits of this program, a number of unknowns remain. Specifically, the cluster set design may be different conceptually to what individuals may perceive in considering participation in a more “traditional” resistance exercise program. As such, it is unclear if this will be a feasible approach to delivering resistance exercise, particularly using the hybrid model of in-person and virtual sessions. Moreover, individuals may not be comfortable with researchers coming to their home to deliver the intervention. We have attempted to address this by offering the alternative of a mutually agreed-upon, safe location. However, we will track reasons for non-participation with a particular focus on comfortability with home visits with the intention that these results may guide the design of similar interventions in the future.

At the completion of this study, we expect to have gathered critical information on the feasibility of a hybrid delivery of cluster set RT to individuals previously treated for NSCLC. Our assessments of recruitment, retention, fidelity, and acceptability will provide important information on whether progression to a randomized controlled trial is warranted. Further, qualitative exit interviews will provide us with important information regarding elements of the trial that need to be revised in accordance with participant feedback.

## Data Availability

n/a
